# Metalens Eyepiece for 3D Holographic Near-Eye Display

**DOI:** 10.3390/nano11081920

**Published:** 2021-07-26

**Authors:** Chang Wang, Zeqing Yu, Qiangbo Zhang, Yan Sun, Chenning Tao, Fei Wu, Zhenrong Zheng

**Affiliations:** 1 State Key Lab of Modern Optical Instrumentation, College of Optical Science and Engineering, Zhejiang University, Hangzhou 310027, China; 11530032@zju.edu.cn (C.W.); 22060564@zju.edu.cn (Z.Y.); 3170102974@zju.edu.cn (Q.Z.); 12130016@zju.edu.cn (Y.S.); 21630032@zju.edu.cn (C.T.); 2 Beijing LLVision Technology Co., Ltd., Beijing 100000, China; wufei@llvision.com

**Keywords:** metalens, computer-generated hologram, three-dimensional near-eye display

## Abstract

Near-eye display (NED) systems for virtual reality (VR) and augmented reality (AR) have been rapidly developing; however, the widespread use of VR/AR devices is hindered by the bulky refractive and diffractive elements in the complicated optical system as well as the visual discomfort caused by excessive binocular parallax and accommodation-convergence conflict. To address these problems, an NED system combining a 5 mm diameter metalens eyepiece and a three-dimensional (3D), computer-generated holography (CGH) based on Fresnel diffraction is proposed in this paper. Metalenses have been extensively studied for their extraordinary capabilities at wavefront shaping at a subwavelength scale, their ultrathin compactness, and their significant advantages over conventional lenses. Thus, the introduction of the metalens eyepiece is likely to reduce the issue of bulkiness in NED systems. Furthermore, CGH has typically been regarded as the optimum solution for 3D displays to overcome limitations of binocular systems, since it can restore the whole light field of the target 3D scene. Experiments are carried out for this design, where a 5 mm diameter metalens eyepiece composed of silicon nitride anisotropic nanofins is fabricated with diffraction efficiency and field of view for a 532 nm incidence of 15.7% and 31°, respectively. Furthermore, a novel partitioned Fresnel diffraction and resample method is applied to simulate the wave propagations needed to produce the hologram, with the metalens capable of transforming the reconstructed 3D image into a virtual image for the NED. Our work combining metalens and CGH may pave the way for portable optical display devices in the future.

## 1. Introduction

Metasurfaces are arrays of subwavelength dielectric or metallic antennas and have been widely investigated for their remarkable capabilities to manipulate the phase, amplitude, and polarization state of the incident light [1]. Due to their extraordinary performances in wavefront shaping, metasurfaces have been used to miniaturize various traditional optical elements into ultrathin devices, such as metalens [2,3,4,5,6,7], meta-hologram [8,9], vortex beam generator [10,11], and wave plate [12,13]. Among those applications, metalenses are an important subcategory of metasurfaces owing to their potential replacement of conventional, bulky optical lenses with their high performance and compactness.

Near-eye display (NED) systems for virtual reality (VR) and augmented reality (AR) show considerable promise as the next generation of display technology; however, the realization of a high sense of immersion, the core requirement of VR/AR devices [14], is impeded by the bulky refractive and diffractive elements in their complicated optical systems, as well as the visual discomfort caused by excessive binocular parallax and accommodation-convergence conflicts [15]. To tackle those problems, thin optical eyepieces such as metalenses [14,16], diffractive optical elements (DOE) [17], and holographic optical elements (HOE) [18] have been investigated for their ability to miniaturize optical systems, and 3D computer-generated holography (CGH) was shown to have the ability to overcome the accommodation-convergence conflicts caused by binocular parallax displays [19]. However, research work integrating ultrathin optical metalens eyepiece with 3D-CGH display technology has not yet been implemented to solve both key limitations of the current NED systems.

In this paper, a display prototype system using a transparent metalens combining Fresnel-diffraction-based 3D-CGH for NED (MCGH-NED) is proposed and experimentally realized. The work initially achieved a miniaturization of the optical system into the ultrathin platform while simultaneously solving the accommodation-convergence conflict of the current NED systems. To this end, first, a 5 mm diameter metalens composed of silicon nitride (Si_3_N_4_) nanofin arrays on a quartz substrate was positioned directly in front of the human eye, operating at 532 nm incidences with high transmission, sufficient modulation efficiency, and a 31° field of view (FOV). In addition, an enlarged 3D virtual image could be viewed after placing the previously mentioned 3D holographic reconstructed image (HRI) in front of the metalens eyepiece within its focal length. The 3D-CGH was implemented using a novel partitioned Fresnel diffraction and resample method based on layer-based methods [20,21]. Furthermore, the HRI was set left-circularly polarized (LCP) since the proposed metalens utilizes the Pancharatnam–Berry (PB) phase manipulation and can converge a circularly polarized (CP) incident light into an output focusing light with opposite helicity [1] so that the output light can only be viewed by adding a right-circularly polarized (RCP) filter, making the proposed NED a VR display system. This work unlocks the potential of metalens and CGH display for future NED display technologies, and applications of this MCGH-NED prototype are envisioned in particular for advanced optical displays, computer vision, wearable devices, etc.

## 2. Materials and Methods

**Figure 1:**
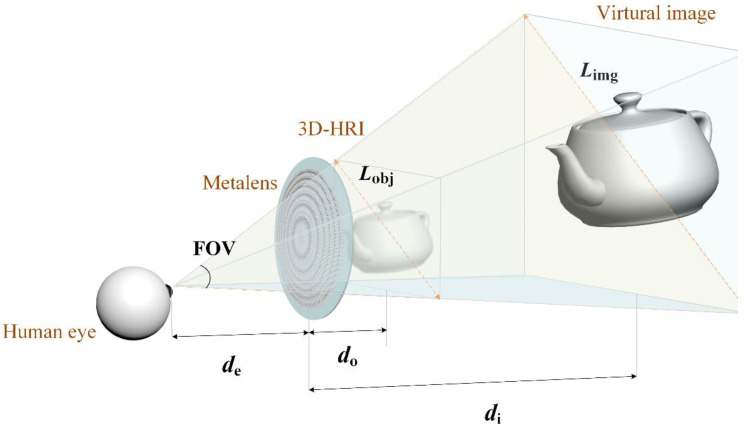
Schematic illustration of the proposed MCGH-NED system. An enlarged virtual image can be viewed by the human eye after placing the transparent HRI in front of the metalens eyepiece.

The basic working principles of the proposed MCGH-NED system that enable the transparent metalens to act as an eyepiece can be illustrated as follows. As shown in Figure 1, the transparent metalens is placed in front of the human eye, and the 3D-HRI, as the imaging object, is placed within the focal length, which is denoted by *f*, of the metalens eyepiece, where *L*_obj_ is the object size, *L*_img_ is the virtual image size, *d*_e_ is the eye relief distance, *d*_o_ is the object distance, and *d*_i_ is the virtual image distance. For this singlet lens imaging system, *d*_o_ and *d*_i_ can be set free as long as *d*_i_ is smaller than the focal length of the metalens. Then, the virtual image is modulated by the metalens eyepiece, floated to the desired location, and imaged onto the human eye or camera, where the displayed virtual image may be enlarged as needed. Thus, the FOV of the system is determined by the eyepiece size as well as the eye relief distance, rather than by the display size, which results in the metalens eyepiece being superior to conventional eyepieces [16], and the corresponding FOV can be expressed as 2 tan^−1^ [*A*_m_/(2*d*_e_)], where *A*_m_ is metalens eyepiece aperture. Furthermore, the largest display object size of the 3D-HRI is decided by a FOV such as *fA*_m_/*d*_e_. It is clear from the above analysis that the desired HRI display size and the image position are linear to *f*, and the FOV of the device is directly related to *A*_m_, so the transparent metalens eyepiece should have a relatively short focal length and large aperture, which means a large numerical aperture (NA).

### 2.1. Design and Fabrication of the Si_3_N_4_ Metalens

Conventional high NA objects composed of complicated compound lenses are essential in imaging, microscopy, spectroscopy, etc., but are bulky and expensive. Thus, singlet planar lenses with high NA in the visible range are in great demand, particularly compared with planar lenses made by diffractive components whose constituent structures are of wavelength scale and would therefore prevent the accurate phase profile that is of vital importance for high NA and efficiency [2]. Metalenses, planar lenses implemented by metasurfaces, are feasible to realize the designed phase profiles accurately as their constituent structures, or unit cells, are of subwavelength scale; hence, high NA can theoretically be achieved by metalenses.

**Figure 2:**
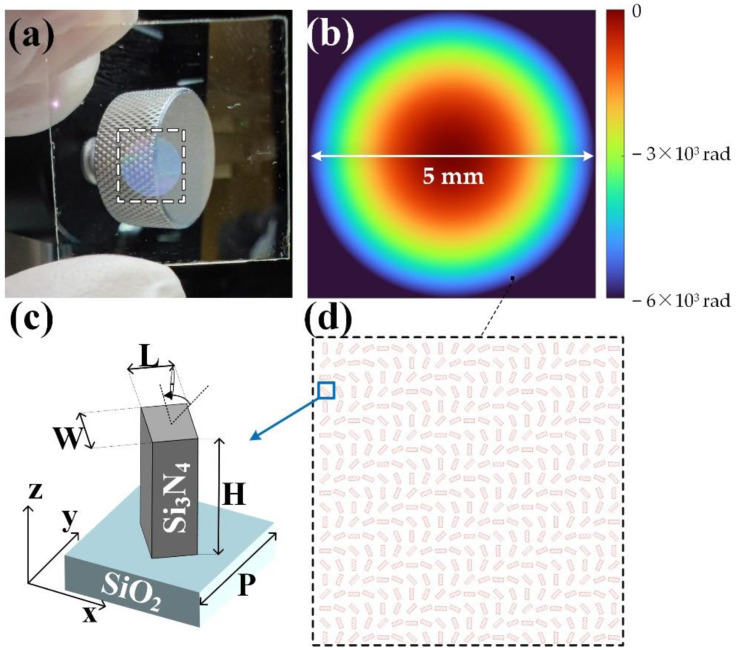
(**a**) The appearance of the fabricated transparent Si_3_N_4_ metalens eyepiece. (**b**) The phase profile of the designed metalens. (**c**) Si_3_N_4_ nanofin on quartz substrate as metalens unit cell. (**d**) Top view of local patterns of the nanofins, rotating to realize the desired PB phases in (**b**).

Optical phase discontinuity theory, derived from generalized laws of reflection and refraction [1], forms the mechanism of phase profile design for metalenses, and requires a converging phase compensation imparted on the metalens interface. The singlet metalens functions like a spherical lens, and normally its phase profile should be of a hyperboloidal form as

(1) $$\varphi (x,y)=2\pi n-\frac{2\pi }{\lambda }\left(\sqrt{{\left(x-f_{x}\right)}^{2}+{\left(y-f_{y}\right)}^{2}+f_{z}^{2}}-f\right),$$

where (*x*, *y*) represents the horizontal position along the metalens interface in the Cartesian coordinates, (*f*_*x*_, *f*_*y*_, *f*_*z*_) is the position of the designed focal point, *f* is the root of quadratic sum of *f*_*x*_, *f*_*y*_ and *f*_*z*_, denoting the focal length, *λ* is the design wavelength, and *n* is an arbitrary integer. In this design, the focal length and the position of the focal point are set to be 6 mm and (0, 0, 6 mm), respectively, and the diameter of the metalens aperture is 5 mm, indicating an NA of 0.4. In addition, the corresponding phase profile that should be imparted along the metalens interface according to Equation (1) is shown in Figure 2b.

There are several phase manipulation mechanisms for realizing phase discontinuity in metalens, for instance, PB phase manipulation, propagation phase manipulation, plasmonic resonance tuning, etc. PB phase manipulation was selected for the metalens design because of its high accuracy of phase compensation realization [1], since it operates for CP incidences and realizes local phase shift by rotating nanofins of metasurface unit cells, which works similarly to half-wave plates. When a CP beam is incident on the nanofin, the transmitted light can be described by

(2) $$E_{t}=\frac{t_{L}+t_{S}}{2}|\sigma \rangle +\frac{t_{L}-t_{S}}{2}\exp(j2\sigma \theta )|-\sigma \rangle ,$$

where the spin-charge *σ* = 1 represents LCP and *σ* = −1 represents RCP; $|\sigma \rangle ={\left[1+i\sigma \right]}^{T}/\sqrt{2}$ is the unit vector of either CP; *t_L_* and *t_S_* represent the complex transmission coefficients for linear polarized light along longer and shorter optical axes of the nanofin, respectively; and *θ* is its orientation angle along *z* axis. For example, when such a PB metasurface is illuminated by an LCP light (*σ* = 1), the transmitted light consists of two components that are a co-polarized LCP light and a cross-polarized RCP light, and an additional phase shift of 2*θ*, which is proportional only to the orientation angle, is imparted onto the RCP light. Moreover, polarization conversion efficiency (PCE) represents the proportion of the CP incidence that is converted to transmitted light with opposite helicity of polarization state. This efficiency is fundamentally determined by the anisotropic coefficients of *t_L_* and *t_S_*, which are decided by the refractive index of the nanofin material, the structural parameters of the nanofin, and the operation wavelength.

The complex refractive index of the material used for the metalens nanofins is of significance for the PCE of single nanofins and the focusing efficiency of the whole metalens. Si_3_N_4_ is chosen for this design due to its exceptionally low extinction coefficient (*k*), large refractive index (*n*) at visible wavelengths, and CMOS-compatible fabrication [2]. Figure 2c illustrates the selected metalens unit cell, consisting of a Si_3_N_4_ nanofin of high aspect-ratio and a layer of quartz substrate. The height of this nanofin is 400 nm, and the actually measured refractive index has an *n* of 1.99 and *k* of about zero at a 532 nm wavelength after growing a 400 nm thick Si_3_N_4_ layer for fabrication. Next, the optimization of other structural parameters for an improved PCE of the nanofin was carried out by full-wave simulations using a commercial package of Ansys Lumerical FDTD Solutions 2020 R2, with an AMD Ryzen Threadripper 3990X CPU (2.9 GHz) with 256 GB RAM utilized as the corresponding computing platform. For the metalens nanofin simulations, periodic boundary condition was applied in the x-direction and y-direction and perfect matching layer (PML) boundary condition was applied at z-direction, with the incidence being set as a 532 nm collimated LCP light. The corresponding computation time for each nanofin simulation was approximately 13–15 s.

It is worth noting that for the design of the whole metalens, the values of the converging phase function as expressed in Equation (1) were directly applied to each nanofin by PB phase manipulation, without considering the local interactions of neighboring nanofins. For this dielectric metalens, although the induced optical fields would theoretically be highly concentrated inside the dielectric nanofins due to their waveguide-like cavity resonances [22], such local interactions are not entirely negligible and may degrade the overall behavior of the designed metalens to some extent.

### 2.2. Methods of 3D CGH

In computer holography, the relation between the source and the destination planes can be given by Fresnel diffraction, the approximation form of Rayleigh–Sommerfeld diffraction, which is written as

(3) $$U_{2}(x_{2},y_{2})=F^{-1}\left\{F\left[U_{1}(x_{1},y_{1})\right]H\left(f_{x},f_{y}\right)\right\},$$

where *U*_1_(*x*_1_, *y*_1_) and *U*_2_(*x*_2_, *y*_2_) represent the complex amplitude of the source plane and the destination plane respectively. The transfer function in Fresnel diffraction is defined as

(4) $$H\left(f_{x},f_{y}\right)=\exp(jkz)\exp(-j\pi \lambda z(f_{x}{}^{2}+f_{y}{}^{2})),$$

where *z* is the propagation distance, *f_x_* = *x*/(*λz*) and *f_y_* = *y*/(*λz*). The MCGH-NED utilizes a novel partitioned Fresnel diffraction method, whose hologram is spatially segmented into multiple partitions as sub-holograms generated from different images. *L*_sub*x*_ = *L_x_*/*M* and *L*_sub*y*_ = *L_y_*/*N* are defined as the width and the height of sub-holograms, respectively, where *L_x_* and *L_y_* denote the width and height of CGH plane, respectively. *M* and *N* are the number of segments along *x* and *y* directions. Then the denotations of *x_m_* = [*m* − (*M* − 1)/2]∙*L*_sub*x*_ and *y_n_* = [*n* − (*N* − 1)/2]∙*L*_sub*y*_ are derived to represent the center coordinates of each sub-holograms, where *m* is an integer from 1 to *M* and *n* is an integer from 1 to *N*, respectively.

**Figure 3:**
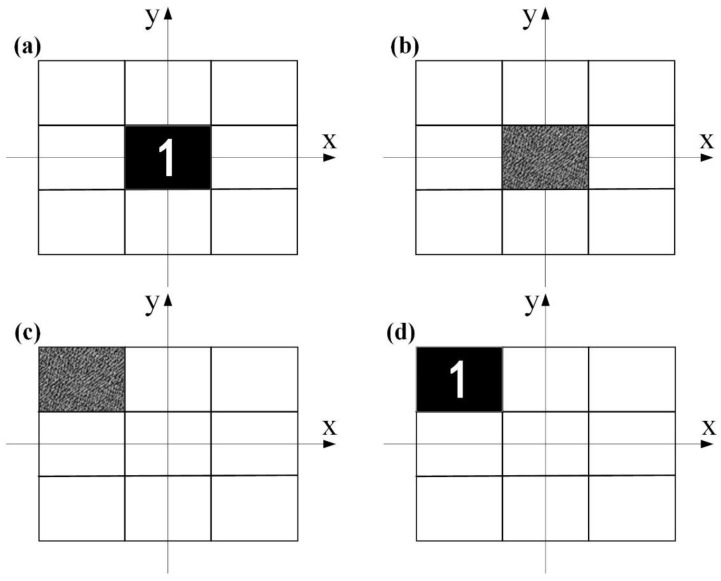
Example of the proposed partitioned Fresnel diffraction method. (**a**,**b**) Original sub-image and the sub-hologram. (**c**) Sub-hologram shifted to another location. (**d**) The reconstructed image after shift.

Figure 3, above, demonstrates an example of the partitioned Fresnel diffraction method. Assuming that *U*_2sub_(*x*_2sub_, *y*_2sub_) in Figure 3a represents the original image of the number “1”, *U*_1sub_(*x*_1sub_, *y*_1sub_) is the sub-hologram produced from *U*_2sub_(*x*_2sub_, *y*_2sub_) as depicted in Figure 3b. When this sub-hologram is shifted to the location of ($x_{m},y_{n}$) as shown in Figure 3c, Equation (3) can be derived as

(5) $$\begin{array}{cc} U\prime_{2\mathrm{sub}}(x_{2\mathrm{sub}},y_{2\mathrm{sub}}) & =F^{-1}\left\{F\left[U_{1\mathrm{sub}}(x_{1\mathrm{sub}}-x_{m},y_{1\mathrm{sub}}-y_{n})\right]H\left(f_{x},f_{y}\right)\right\}\\ & =F^{-1}\left\{F\left[U_{1\mathrm{sub}}(x_{1\mathrm{sub}},y_{1\mathrm{sub}})\right]H\left(f_{x},f_{y}\right)\exp[-j2\pi (f_{x}x_{m}+f_{y}y_{n})]\right\}. \end{array}$$

According to the convolution theorem, Equation (5) can be rewritten as

(6) $$\begin{array}{cc} U\prime_{2\mathrm{sub}}(x_{2\mathrm{sub}},y_{2\mathrm{sub}}) & =U_{2\mathrm{sub}}(x_{2\mathrm{sub}},y_{2\mathrm{sub}})⊗\delta \left(x_{2\mathrm{sub}}-x_{m},y_{2\mathrm{sub}}-y_{n}\right)\\ & =U_{2\mathrm{sub}}\left(x_{2\mathrm{sub}}-x_{m},y_{2\mathrm{sub}}-y_{n}\right), \end{array}$$

where *U′*_2sub_(*x*_2sub_, *y*_2sub_) is the reconstruction of the shifted *U*_1sub_(*x*_1sub_, *y*_1sub_), proving that the reconstruction in Figure 3d is of the same size and location as its sub-hologram in Figure 3c.

**Figure 4:**
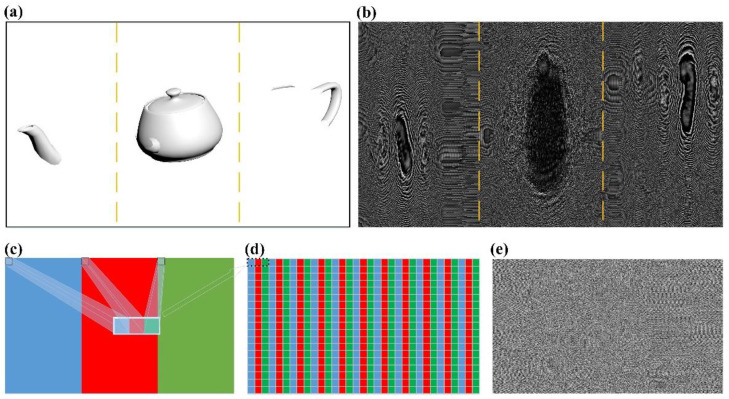
The proposed resample method. (**a**,**b**) The original image consisting of three sub-images sliced from a 3D model “teapot” and the corresponding hologram. (**c**) Three sub-holograms and a three-pixel unit composed of 3 pixels from 3 sub-holograms at the same position. (**d**) Three-pixel units arranged in a side-by-side fashion. (**e**) The hologram generated from (**b**) through the resample method.

For a more complex 3D scene, multiple layers are produced by slicing the 3D scene with various contents at the same position. Harnessing no overlap between different layers in the hologram, the proposed method eliminates the crosstalk between layers that decreases the reconstruction quality in conventional layer-based methods. However, if the reconstructed image shifts with the location of the sub-hologram rather than being at the center, a broken 3D scene would appear. To correct the position of the reconstruction at the center, a resample method is used as explained in Figure 4. In this demonstration, the original image is a 3D model “teapot”, which is then sliced into three layers according to the depth to form 3 sub-images. The hologram in Figure 4b consists of three sub-holograms generated from sub-images in Figure 4a. As Figure 4c depicts, pixels at the same relative positions in 3 sub-holograms are picked to form a three-pixel unit that is set in a side-by-side way in a new hologram as shown in Figure 4d,e.

## 3. Results and Discussion

**Figure 5:**
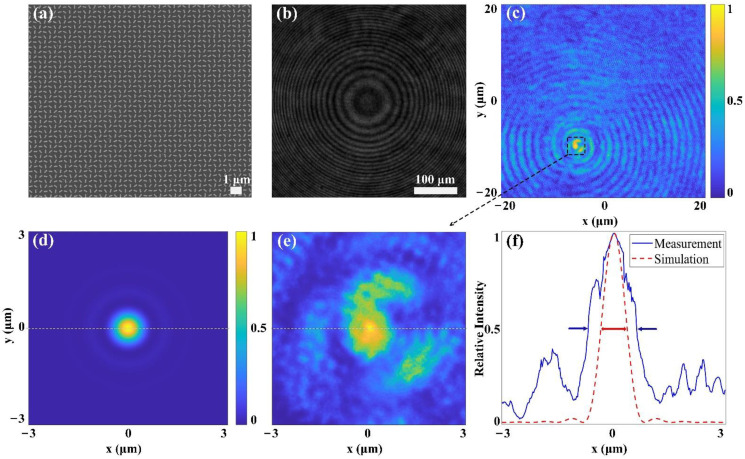
The characterizations of the fabricated singlet metalens. (**a**) A scanning electron micrograph (SEM) image of the local part from the fabricated metalens. (**b**) Optical image of the central part from the fabricated metalens under 532 nm microscopic illuminating light. (**c**) Measured focal spot intensity profile at 532 nm wavelength by a commercial object (100 × OLYMPUS, NA = 0.90). (**d**) Simulated focal spot intensity profile at 532 nm wavelength by FDTD method. (**e**) Magnified image of the measured focal spot intensity profile from (**c**). (**f**) Central focal spot intensity profiles along horizontal axis from (**d**) and (**e**), respectively.

As a result of the optimization for adequate PCE by FDTD simulations, each nanofin has a high aspect ratio and a rectangular cross-section, with longer length *L*, shorter length *W,* and periodic spacing *P* of 300 nm, 105 nm and 400 nm, while the simulated PCE is 24.3% under a 532 nm incident light. The subsequent fabrication processes are carried out as follows: the growth of a 400 nm thick Si_3_N_4_ layer on a double-polished quartz plate is prepared by using plasma-enhanced chemical vapor deposition (PECVD), and subsequently a layer of ZEP-520A e-beam resist is spin-coated on it. This sample is then exposed through electron-beam lithography (EBL) to define the structure patterns, which are revealed after the development process of the resist. Next, a 100 nm thick Cr layer is deposited on the sample as a hard-etching mask by electron gun evaporation, and a lift-off process is followed. After the removal of the resist, the required patterns are transferred to the Cr hard mask, and the final sample is obtained by reactive ion etching (RIE) and the removal of the patterned Cr hard mask. Figure 5, below, shows the characterizing results of the fabricated metalens.

Figure 5a represents the scanning electron micrograph (SEM) image of the fabricated singlet metalens, and Figure 5b shows the microscopic image of the fabricated metalens under 532 nm laser illumination. For Figure 5c, the corresponding full-width at half-maximum (FWHM) measured at 532 nm LCP incidence is 1.49 μm, approximately 2.2 times the diffraction limit (*λ/*(2NA) = 0.67 μm), and the focusing efficiency of the fabricated metalens, defined as the focusing power from a circular area with a diameter of three times FWHM over the incident power, is 15.7% when measured under a 532 nm LCP incidence.

In addition, in order to compare the focusing characteristics between the simulation and the measurement, the simulated electric field intensity distribution of the focal plane for a minimized metalens, which has a relatively small aperture diameter but the same NA of 0.4 as the fabricated metalens, is shown in Figure 5d. In this case, the simulated minimized metalens has an aperture diameter of 60 μm with a focal length of 64 μm, and PML boundary condition is applied at x-direction, y-direction, and z-direction, with the corresponding computation time for such minimized metalens being about 5 h and 18 min. There are two reasons for simulating such a minimized metalens: first, the designed metalens of a 5 mm diameter aperture is composed of over 1.22 × 10^8^ Si_3_N_4_ nanofins, demanding excessive computation resources for FDTD simulations in current computing stations; second, when two metalenses of different apertures but the same NA of 0.4 are illuminated under unchanged incident light, their focal spots and the corresponding diffraction limits will theoretically be identical, indicating the same focusing characteristics.

The simulated focal spot intensity profile of such minimized metalens is shown in Figure 5d, and a comparison of the central focal spot intensity profiles along the horizontal axis from Figure 5d,e, which is a magnified picture of Figure 5c, is illustrated in Figure 5f. The simulated FWHM for such a minimized metalens with the same NA is about 0.83 μm, far smaller than that of the measured FWHM of the fabricated metalens, which is 1.49 μm, as mentioned above. Factors such as fabrication errors and local interactions among neighboring metalens nanofins would all account for the degrading of the behavior of the fabricated metalens.

**Figure 6:**
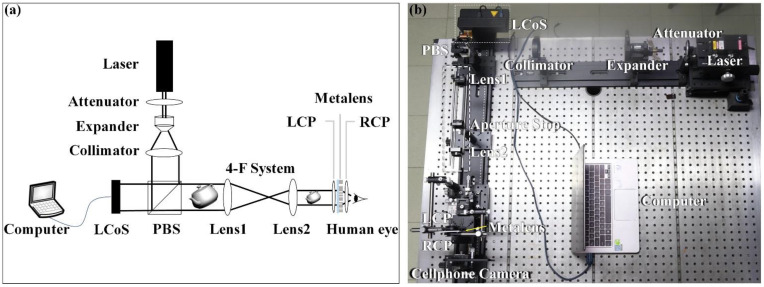
(**a**) Schematic of the MCGH-NED experimental setup. (**b**) Photograph of the MCGH-NED experimental setup.

Furthermore, in order to characterize the performance of the proposed metalens in the 3D holographic display, optical imaging experiments were carried out and the experimental setup for the MCGH-NED system is demonstrated in Figure 6. First, a collimated 532 nm laser beam is deflected by a polarization beam splitter (PBS) and hits a phase-only spatial light modulator (SLM), which is a liquid crystal on silicon (LCoS, Holo-eye LETO), as the image engine. This LCoS has a resolution of 1920 × 1080 with the pixel pitch being 6.4 μm. After the hologram-loaded LCoS is illuminated by the laser source the reconstruction is generated and the 3D HRI transmits through an optical 4-F system, composed of two lenses of 200 mm and 100 mm focal lengths with a pinhole between them. The HRI is thus shrunk by a factor of 0.5 in order to fit the maximum display size of the metalens eyepiece, and the measured eye relief is 9 mm, indicating a FOV of 31°.

As for the problem of zero-order light, the digital blazed grating is introduced in the hologram. It can be expressed as *φ*_bg_(*x*, *y*) = (2π/*T*)∙mod(*cx* + *dy*, *T*), where *T* is the period of digital blazed grating, and *c* and *d* are two constants related to the shift distance in *x* and *y* directions, respectively. For this system, *c* as well as *d* are 0 and 1, and *T* is 1.5 times the sampling interval of the hologram. After the modulation of digital blazed grating, the HRI can be separated from zero-order light, which can be eliminated by the pinhole at the confocal position of the 4-F system for suppressing noise. Ultimately, the transferred 3D HRI is positioned within the focal length of the metalens, and an LCP polarizer is placed between it and the metalens eyepiece, which can focus LCP incidence and output focused RCP light. Having been modulated by the metalens, the output light passes through an RCP polarizer for filtering the desired virtual image and realizing the VR display. The floated virtual image is then viewed by the human eye and captured by an iPhone 6 cellphone camera.

**Figure 7:**
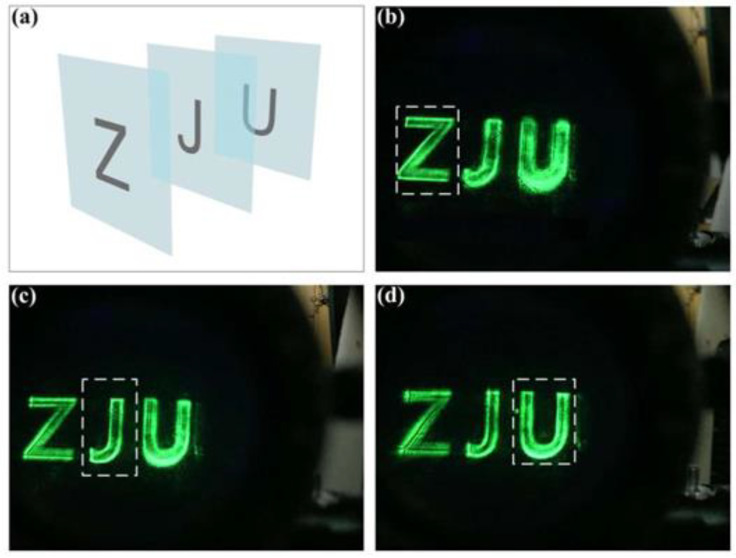
3D near-eye display of ZJU characters. (**a**) The layered model ZJU. (**b**–**d**) The reconstructed images focused at Z, J, and U, respectively.

Figure 7 above shows the experimental results of a 3D holographic near-eye display with the designed metalens as an eyepiece. Figure 7a is the original layered model of ZJU. Three sub-holograms are calculated from Z, J, and U, respectively, with different propagation distances, and the propagation distance interval is set at 100 mm. The images are reconstructed at different distances, and as a result, the characters Z, J, and U are separately focused at corresponding positions, as shown in Figure 7b–d. It can be observed that when one of the characters is focused by the metalens eyepiece, the other two characters are blurred.

**Figure 8:**
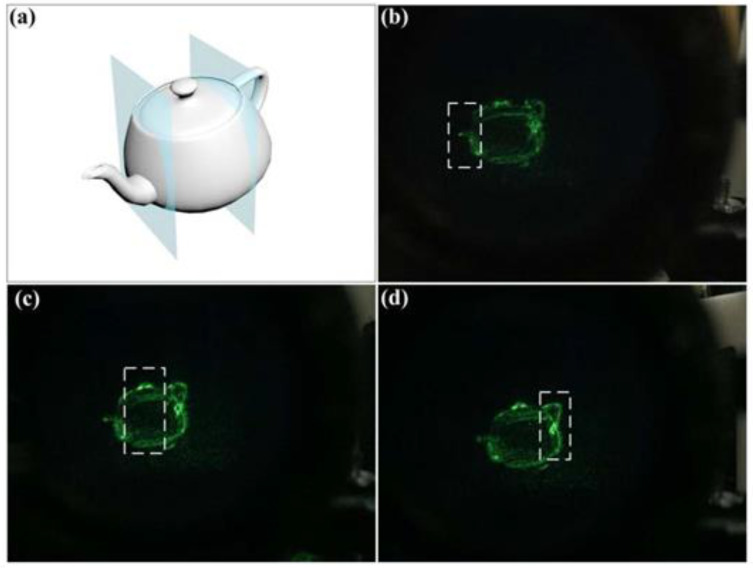
Experimental results of 3D near-eye display of “teapot”. (**a**) The sliced 3D model “teapot”. (**b**–**d**) The reconstruction focused on the spout, lid and handle, respectively.

In Figure 8 below, a complex 3D model, “teapot”, is displayed and observed experimentally. As Figure 8a illustrates, the model is sliced into three layers and the spacing between neighboring layers is 100 mm. Then the holograms are produced from these layers according to the resample method mentioned above, and Figure 8b–d are the captured virtual images of the HRI imaged by the metalens eyepiece. It can be seen that the spout of the teapot is focused in Figure 8b, while the lid and the handle are blurred. In contrast, the lid and the handle in Figure 8c,d are focused respectively, while the remaining two parts are blurred when viewing through the metalens eyepiece.

## 4. Conclusions

In this paper, a holographic 3D-NED system using a transparent metalens as an eyepiece, named MCGH-NED, was proposed. Featuring a subwavelength-scale wavefront manipulation, ultrathin size, and high-performance features, a metalens eyepiece with 15.7% focusing efficiency, a diameter of 5 mm, and an NA of 0.4 was designed and fabricated to overcome the issues of bulkiness and limited FOV in the NED system. Furthermore, a CGH technique developed by a novel partitioned Fresnel diffraction and resample method was utilized to demonstrate the display characteristics of the metalens-based 3D-NED system, confirming the advance of 3D-CGH display over traditional binocular NED displays. Experimental results demonstrated a 31° FOV of the MCGH-NED system and clear 3D image display performance of the metalens as an eyepiece for observing the 3D-CGH virtual images. Furthermore, the CP light-converting property of the proposed metalens eyepiece realizes the VR display mode. This work takes the initiative in integrating metalens and 3D-CGH displays in NED systems, and may hopefully find wide application in advanced and compact optical display technologies in the near future.

## Data Availability

No new data were created or analyzed in this study. Data sharing is not applicable to this article.
